# Differential Gene Expression in the Nucleus Accumbens and Frontal Cortex of Lewis and Fischer 344 Rats Relevant to Drug Addiction

**DOI:** 10.2174/157015911795017290

**Published:** 2011-03

**Authors:** A Higuera-Matas, G. L Montoya, S.M Coria, M Miguéns, C García-Lecumberri, E Ambrosio

**Affiliations:** Departamento de Psicobiología, Facultad de Psicología, UNED, Madrid, Spain

**Keywords:** Drug addiction, gene expression, genetic differences, Fischer 344, Lewis, microarrays.

## Abstract

Drug addiction results from the interplay between social and biological factors. Among these, genetic variables play a major role. The use of genetically related inbred rat strains that differ in their preference for drugs of abuse is one approach of great importance to explore genetic determinants. Lewis and Fischer 344 rats have been extensively studied and it has been shown that the Lewis strain is especially vulnerable to the addictive properties of several drugs when compared with the Fischer 344 strain. Here, we have used microarrays to analyze gene expression profiles in the frontal cortex and nucleus accumbens of Lewis and Fischer 344 rats. Our results show that only a very limited group of genes were differentially expressed in Lewis rats when compared with the Fischer 344 strain. The genes that were induced in the Lewis strain were related to oxygen transport, neurotransmitter processing and fatty acid metabolism. On the contrary genes that were repressed in Lewis rats were involved in physiological functions such as drug and proton transport, oligodendrocyte survival and lipid catabolism.

These data might be useful for the identification of genes which could be potential markers of the vulnerability to the addictive properties of drugs of abuse.

## INTRODUCTION

Drug addiction arises from the interplay between social and biological factors. Among the later, genetic variables are thought to play a major role [1, 2]. This fact has been corroborated in animal studies, especially in those involving Lewis (LEW) and Fischer 344 (F344) rats. These two rat strains show differential responses to both drugs of abuse and stressors [3]. LEW rats are more sensitive to the reinforcing properties of cocaine, morphine or ethanol and they faster acquire the self-administration of these drugs [4-9].

On the other hand, LEW and F344 rats differ in several neurochemical parameters which are related to reward processes. For instance, LEW animals have higher levels of tyrosine hydroxylase in the ventral tegmental area (VTA) but lower levels in the nucleus accumbens (NAcc) when compared with F344 rats [10-14]. There are also differences in the levels of µ opioid receptors in several brain regions between both strains and these proteins are differentially modulated after morphine self-administration and during the extinction of this behavior [8]. Moreover, it has been shown that there are higher levels of dopamine in the NAcc-Core of LEW rats after acute administration of several drugs of abuse [15].

Another important difference between both strains is the reactivity of the hypothalamic-pituitary-adenal (HPA) axis which displays a higher activity in the F344 strain when compared with the LEW strain [3, 16, 17].

Notwithstanding all the data referenced above, the differential gene expression pattern in areas of the reward circuit of these two strains was unexplored. Therefore we designed the following experiment where we used Affymetrix cDNA microarrays to study gene expression in two key components of the reward system, the NAcc and frontal cortex (FC) [18] of the addiction-prone LEW strain and its histocompatible control the F344 strain.

## MATERIALS AND METHODS

### Animals

Male F344 (n=9) and LEW (n=9) rats weighing 300-320 g at the beginning of the experiments were used.

All animals were maintained at a constant temperature (20±2º C) in a 12 hours light-dark cycle (lights on at 08:00 hours), with free access to food and water (commercial diet for rodents A04/A03; Panlab, Barcelona, Spain). All animals were maintained and handled according to European Union Laboratory Animal Care Rules (86/609/EEC Directive).

### RNA Extraction

Two weeks after arrival in the vivarium, the rats were lightly anesthetized with isoflourane and decapitated. Brains were quickly removed and the FC and NAcc dissected out on ice. After weighed, the tissue was preserved in RNAlater solution (Qiagen, United Kingdom) at 4ºC overnight and then at -20ºC until RNA extraction. All the procedures were performed under the maximum sterility and dissection instruments and work surfaces were thoroughly cleaned with RNAase Zap (Ambion, Spain) to prevent RNAase activity. Total RNA extraction was performed with RNAeasy extraction kit with in-column DNAase digestion (Qiagen, United Kingdom), according to manufacturer’s instructions. RNA quantity was determined by 260 nm absorbance and integrity was determined with an Agilent Bioanalyzer 2100 (all samples showing a RIN >8).

### Microarray Analysis

For microarray studies we made 3 pools of RNA per structure and strain, resulting on 12 different pools of RNA (3 for NAcc of LEW rats, 3 for frontal cortex of LEW rats, 3 for NAcc of F344 rats and 3 for frontal cortex of F344 rats). For each strain, RNA from 9 rats was used.

Probe preparation, hybridization and analysis were carried out in the genomics core facility of the “Universidad Complutense de Madrid”. Probes for the Affymetrix Gene 1.0 ST were prepared and hybridized to the array using the “GeneChip Whole Transcript Sense Target Labeling Assay” (Affymetrix) according to the manufacturer’s suggestions. Briefly, for each sample of 200 ng of total RNA, cDNA was synthesized with random hexamers tagged with a T7 promoter sequence. The double-stranded cDNA was used as a template for amplification with T7 RNA polymerase to create antisense cRNA. Next, random hexamers were used to reverse transcribe the cRNA to produce single-stranded sense strand DNA. The DNA was fragmented and labeled with terminal deoxynucleotidyl transferase. The probes from each pool were hybridized to the Affymetrix Gene 1.0 ST array for 16 h at 45ºC. Subsequently, arrays were scanned and gene expression indexes were calculated with the RMA software [19].

Expression ratios higher than 2 were considered to reflect induction of gene expression whereas ratios lower than 0.5 were taken as indexes of inhibition of expression. Significance was set to p<0.05.

## RESULTS

For the sake of clarity, we have organized the data according to induction/inhibition and anatomical localization criteria (induction/inhibition only in the NAcc, only in the FC or in both) (See Tables **1**-**6**).

Only a limited number of the genes studied were significantly different between strains. LEW rats showed higher expression in both the NAcc and FC in only 5 of the genes included in the array. These genes were related to different functions such as protein transport, lipid metabolism, nitrogen metabolism, hydrolase activity or organismal development (Table **1**). 

The number of genes that were significantly induced in the FC and not in the NAcc was higher in the LEW strain compared with the F344 strain. Two of these genes were related to oxygen transport (LOC689064 and MGC72973) and another pair was related to ion transport (Atp5g2 and Slc17a6, the latter being also involved in neurotransmitter uptake). The rest of the genes induced in the FC were involved in different biological processes such as ADP-ribosylation (Adprhl1), regulation of cell cycle (RGD1310778) and signal transduction (Gpr103) to mention just a few examples (see Table **2** for further details).

As for the NAcc, only a few genes were induced in this structure in the LEW strain, without alteration in FC expression patterns. These genes were involved in sodium transport (Slc10a4), sphingolipid metabolism (Fa2h_predicted//Wdr59), cytokinesis (Anln), organismal development (Hydin) and apoptosis (Perpr) (see Table **3**).

With regard to inhibited expression, the genes that were repressed in both the NAcc and FC in LEW rats compared with F344 were more numerous than those which were induced. Interestingly, two of these genes (Nqo2 and Akr1b10) were involved in oxidation-reduction processes while the rest of the inhibited genes had diverse functions, such as calcium binding (Pvalb), sphingolipid metabolism (Sgms2) or drug transport (Abcg2) among others (see Table **4**).

Specific gene expression inhibition in the FC comprised more genes, with a broader range of functions. More specifically, three of the genes that were inhibited in the FC but not in the NAcc were related to neurotransmitter regulation (transport, secretion/exocytosis: Sv2c, Unc13c and Sytl5 respectively) and two of them were involved in organismal development (Dlx5 and Cml2). The others were related to biological processes such as membrane organization (Ap1s2), regulation of DNA damage (Chd1l), regulation of oligodendrocyte prolongations (Ermn) and apoptosis (Alox15) among others (Table **5**).

Lastly, the genes inhibited in the NAcc but not in the FC were less in number and related to several functions, for example neurotransmitter transport (Slc6a20), cell growth (Igfbp2 and Igfbp6), DNA replication (Rad1) or proton transport (ATP8).

## DISCUSSION

In this work we have used cDNA microarrays analysis to study the differential gene expression profile in the NAcc and FC of the addiction-prone LEW strain and its histocompatible control the F344 strain. Although further real-time PCR studies must now validate these preliminary results, it seems that there are four set of genes differentially expressed in both inbred rat strains.

### 1. Induced Genes

#### 1.1. Genes that were Induced in Both the NAcc and FC in LEW Rats as Compared with F344 Rats

To the best of our knowledge, neither of the genes that were induced in LEW rats as compared with F344 rats in both the NAcc and FC had any known relationship to drug addiction and were mainly involved in general homeostatic processes.

#### 1.2. Genes that were Induced in the FC but not in the NAcc as Compared with F344 Rats

Within the genes that were induced in the FC but not in the NAcc of LEW rats compared with F344 animals, Atp5g2 could be related to drug addiction phenomena since it has been shown to be up-regulated in the pancreatic cells of alcohol-consuming rats, a fact which parallels with mitochondrial damage [20]. In nerve tissue, the up-regulated expression of this gene in the FC of LEW rats could also correlate with an enhanced sensitivity to alcohol-induced damage in LEW rats, a possibility which has not been explored as yet. The beta-globin gene (LOC 689064ç9 was also induced in the FC and not in the NAcc. An interaction between ethanol and beta-globin has been reported [21] since it has been shown that acetaldehyde, the major metabolite of ethanol forms adducts with the beta-globin chain of hemoglobin, which are typically used as a marker of ethanol consumption [21]. Although the functional implications of the increased expression of the beta-globin gene are not clear, it could be related to the stronger sensitivity to ethanol effects observed in the LEW strain [9]. Slc17a6 gene (which codes a vesicular glutamate transporter protein) was also induced in the FC of LEW rats when compared with F344 rats. Interestingly, this gene was found to be up-regulated in the VTA by extended alcohol and/or tobacco abuse in humans [22], suggesting a role for this gene in the enhanced sensitivity to alcohol reported in the LEW strain.

ADP-ribosylation is a major mechanism for G-protein inactivation. In fact, G proteins have been shown to be altered in opiate-dependent patients [23] and G_i_ protein inactivation by pertussis-toxin-induced ADP-ribosylation is able to reverse some of the behavioral responses elicited by dopaminergic agents during cocaine withdrawal [24]. As regards this, the increased expression of the Adprhl1 gene (which de-ADP ribosylates G proteins) in the FC of LEW rats could be a compensatory mechanism for an increased ADP-ribosylation activity. This enhanced ADP-ribsylation could be related to altered withdrawal syndromes in these two strains.

The rest of the genes induced in the FC but not in the NAcc have either unknown functions or no clear relationship with drug addiction processes.

#### 1.3. Genes that were Induced in the NAcc but not in the FC of LEW Rats as Compared with F344 Rats

The next set of genes comprises those induced in the NAcc but not in the FC. Among these, one gene that merits mention here is the apoptosis-related gene Perp which is induced in degenerating dopamine neurons [25]. Given that dopaminergic toxicity has been sometimes associated to enhanced sensitivity to rewarding effects of several drugs [26, 27], it is tempting to speculate that Perp enhanced expression in the NAcc of LEW rats could be related to higher toxicity following a dopaminergic insult resulting in augmented sensitivity to the rewarding properties of drugs such as methamphetamine or ethanol. Nonetheless, this hypothesis has not been experimentally tested yet. The rest of the genes in this set have no clear relationship with drug addiction or reward.

### 2. Repressed Genes

#### 2.1. Genes that were Repressed in the NAcc and FC in LEW Rats as Compared with F344 Rats

Among the genes that were inhibited in both the FC and NAcc of LEW rats compared with F344 animals, Pvalb is of special interest given that it is a marker for GABAergic neurons and there are several reports in the literature showing that drugs of abuse alter the number of parvalbumin-containing GABAergic neurons in several areas of the brain [28, 29]. Another interesting gene which was inhibited in both structures was Sgms2, which codes sphingomyelin synthase 2. Deficiency of this enzyme has been related to attenuated NFκB activation [30] which is a transcription factor involved in different aspects of drug addiction [31, 32]. Another gene which was inhibited was Nqo2 which is involved in oxidation-reduction reactions and has been associated with increased risk of methamphetamine-induced psychosis [33]. Interestingly, this gene is also implicated in plasticity mechanisms regulating learning and memory [34], processes that are also playing a central role in addictive behaviors [35, 36]. The other genes in this set have no clear relationship with drug addiction or reward processes.

#### 2.2. Genes that were Repressed in the FC but not in the FC of LEW Rats as Compared with F344 Rats

We then examined the genes that were inhibited in the FC but not NAcc of LEW rats as compared with F344 rats. In this set of genes, Sv2c, the gene coding for synaptic vesicle glycoprotein 2c was repressed. This protein binds to synaptotagmin and regulates exocytosis [37], a process that is involved in normal synaptic transmission and in drug addiction [38]. This is in accordance with the fact that Sytl5, the gene that codes the synaptotagmin-like 5 and which is also involved in neurotransmitter secretion, is down-regulated. Interestingly, the Unc13c gene was inhibited in the FC of LEW and this gene is also known for regulating neurotransmitter secretion (Table **5**). Therefore, three neurotransmitter release-related genes seem to be affected in the LEW strain. The relevance of this fact to normal synaptic function and addiction phenomena remains to be determined. The expression of Mme gene, which codes for the membrane metallo endopeptidase (enkephalinase) enzyme, was repressed in the FC but not the NAcc of LEW rats. This enzyme is responsible for the degradation of several endogenous peptides including the enkephalins [39]. A lower expression of this enzyme would result in high enkephalin levels which are observed after ethanol [40] or morphine [41] injections. Additionally, high levels of met-enkephalins are also responsible for attenuated withdrawal responses during opiate withdrawal [42]. Chd1l gene was also repressed in the FC of LEW rats. The protein coded by this gene (chromodomain helicase DNA binding protein 1-like) is able to interact with Nur77 and inhibit its translocation from the nucleus to the mitochondria [43]. Nur77 is an apoptosis-related protein involved in amphetamine-induced locomotion [44] and cocaine self-administration [45]. Interestingly, chronic cocaine upregulated the levels of NGFI-B/Nur77 family of nuclear orphan receptors in F344 rats while no effect was observed in LEW rats [46], highlighting differences in the dynamics of the expression of this gene in both strains in basal conditions as well as after drug challenges. Therefore, high levels of Nur77 (resulting from reduced Chd1l activity) could contribute to explain the enhanced susceptibility to drug self-administration of the LEW strain. The rest of the genes of this set have no clear relationship to reward or drug addiction processes.

#### 2.3. Genes that were Repressed in the NAcc but not in the FC of LEW Rats as Compared with F344 Rat

Lastly, we found a reduced set of genes that were inhibited in the NAcc but not FC of LEW rats as compared with F344 rats. ATP8 (coding subunit 8 of the ATP synthase enzyme) inhibition could have some importance in explaining the vulnerability to the addictive properties of drugs of abuse. In this sense, it has been already found that the alpha-subunit of ATP synthase is differentially modulated in two subsets of rats which differed in the extinction of cocaine-induced conditioned place preference [47]. Another couple of interesting genes was Igfbp6 and Igfbp2 which have been shown to be involved in mood disorders [48-50] and therefore could also have relevance to drug addiction given the interrelationship between both psychopathological spectra [51, 52]. The rest of the genes in this set have no direct or clear relationship to drug addiction.

In conclusion, we have found four set of genes (each one only including a limited number of examples) that may be useful markers of vulnerability to addiction. Further studies should validate these results and test the implication of each of the genes reported here in addiction-related phenomena.

## Figures and Tables

**Table 1:** Genes that were Induced Both in the NAcc and FC in LEW Rats as Compared with F344 Rats

Gene Symbol	Gene Description	mRNA Accession No.	Biological Process
**Pitpnm1 **	phosphatidylinositol transfer protein, membrane-associated 1	NM_001008369	protein transport
**Acsm3 **	acyl-CoA synthetase medium-chain family member 3	NM_033231	lipid metabolic process
**Nit2 **	nitrilase family, member 2	NM_001034126	nitrogen compound metabolic process
**RGD1309362 **	similar to interferon-inducible GTPase	BC098065	hydrolase activity, acting on acid anhydrides
**RGD1561619_predicted **	similar to Camello-like 2 (predicted)	XM_001074225	multicellular organismal development

**Table 2:** Genes that were Induced in the FC but not in the NAcc of LEW Rats as Compared with F344 Rats

Gene Symbol	Gene Description	mRNA Accession No.	Biological Process
**Atp5g2 **	ATP synthase, H+ transporting, mitochondrial F0 complex, subunit C2 (subunit 9)	NM_133556	ion transport (proton transport)
**LOC689064 **	beta-globin	NM_001111269	oxygen transport
**MGC72973 **	beta-glo	NM_198776	oxygen transport
**RGD1563482 **	similar to hypothetical protein FLJ38663	BC168187	unknown
**RGD1310778 **	similar to Putative protein C21orf45	BC167102	cell cycle
**LOC363306 **	hypothetical protein LOC363306	ENSRNOT00000041659	unknown
**Slc17a6 **	solute carrier family 17 (sodium-dependent inorganic phosphate cotransporter), member 6	NM_053427	Ion transport /neurotransmitter uptake)
**Ccdc95 **	coiled-coil domain containing 95	NM_001013900	unknown
**Ccdc77 **	coiled-coil domain containing 77	ENSRNOT00000056200	unknown
**Adprhl1 **	ADP-ribosylhydrolase like 1	NM_001013054	protein amino acid de-ADP-ribosylation/protein amino acid de-ADP-ribosylation
**RGD1307225 **	similar to MEGF6	NM_001107663	unknown
**Gpr103 **	G protein-coupled receptor 103	NM_198199	signal transduction/G-protein coupled receptor protein signaling pathway
**LOC692042 **	hypothetical protein LOC692042	XM_001081377	unknown
**LOC300308 **	similar to hypothetical protein 4930509O22	BC090074	protein amino acid phosphorylation
**LOC688778 **	similar to fatty aldehyde dehydrogenase-like	ENSRNOT00000024034	oxidation reduction/cellular aldehyde metabolic process

**Table 3:** Genes that were Induced only in the NAcc but not in the FC of LEW Rats as Compared with F344 Rats

Gene Symbol	Gene Description	mRNA Accession No.	Biological Process
**Slc10a4 **	solute carrier family 10 (sodium/bile acid cotransporter family), member 4	NM_001008555	ion transport(sodium)
**Fa2h_predicted // Wdr59 **	fatty acid 2-hydroxylase (predicted) // WD repeat domain 59	ENSRNOT00000025625	fatty acid biosynthetic process (sphingolipid metabolism)
**Tmem63a // Tmem6 **	transmembrane protein 63a // transmembrane protein 6	ENSRNOT00000004519	unknown
**Anln **	anillin, actin binding protein (scraps homolog, Drosophila)	ENSRNOT00000024361	cytokinesis
**Hydin **	hydrocephalus inducing	XM_226468	multicellular organismal development
**LOC365476 **	similar to chromosome 10 open reading frame 79	XM_345041	unknown
**RGD1559942 **	similar to hypothetical protein	ENSRNOT00000044984	Unknown
**Perp **	PERP, TP53 apoptosis effector	NM_001106265	induction of apoptosis

**Table 4:** Genes that were Inhibited both in the NAcc and FC of LEW Rats as Compared with F344 Rats

Gene Symbol	Gene Description	mRNA Accession No.	Biological Process
**Myo5c **	myosin VC	NM_001108167	secretory granule trafficking
**Pvalb **	parvalbumin	NM_022499	calcium ion binding
**LOC679726 **	similar to spermatogenesis associated glutamate (E)-rich protein 4d	ENSRNOT00000040473	unknown
**Fcrls **	Fc receptor-like S, scavenger receptor	NM_001107702	scavenger receptor activity
**Thoc4 **	THO complex 4	NM_001109602	nuclear mRNA splicing, *via* spliceosome
**Sucnr1 **	succinate receptor 1	NM_001001518	signal transduction (G-protein coupled receptor signaling)
**LOC686123 **	similar to leucine rich repeat and coiled-coil domain containing 1	ENSRNOT00000059442	protein binding
**Sgms2 **	sphingomyelin synthase 2	NM_001014043	fatty acid biosynthetic process (sphingolipid metabolism)
**Abcg2 **	ATP-binding cassette, sub-family G (WHITE), member 2	NM_181381	drug transport
**Nqo2 **	NAD(P)H dehydrogenase, quinone 2	NM_001004214	memory formation/ oxidation reduction
**Akr1b10 **	aldo-keto reductase family 1, member B10 (aldose reductase)	NM_001013084	oxidation reduction
**Ccdc42 **	coiled-coil domain containing 42	NM_001107009	unknown

**Table 5:** Genes that were Inhibited in the FC but not in the NAcc of LEW Rats as Compared with F344 Rats

Gene Symbol	Gene Description	mRNA Accession No.	Biological Process
**Nxph4 **	neurexophilin 4	NM_021680	neuropeptide-like activity
**Slc35d3 **	solute carrier family 35, member D3	NM_001107522	unknown
**Sv2c **	synaptic vesicle glycoprotein 2c	NM_031593	neurotransmitter transport
**Unc13c **	unc-13 homolog C (C. elegans)	NM_173146	regulation of neurotransmitter secretion
**Lrrc1 **	leucine rich repeat containing 1	NM_001014268	protein binding
**Sytl5 **	synaptotagmin-like 5	NM_178333	exocytosis
**Dlx5 **	distal-less homeobox 5	NM_012943	multicellular organismal development/nervous system development
**Lpl **	lipoprotein lipase	NM_012598	lipid catabolic process/fatty acid biosynthetic process
**Ap1s2 **	adaptor-related protein complex 1, sigma 2 subunit	NM_001127531	intracellular protein transport/membrane organization
**Mme **	membrane metallo endopeptidase	NM_012608	proteolysis
**Ermn **	ermin, ERM-like protein	NM_001008311	regulation of cell projection organization(oligodendrocites)
**Dpyd **	dihydropyrimidine dehydrogenase	NM_031027	oxidation reduction/ purine and pyrimidine base catabolic processes
**Cml2 **	Camello-like 2	NM_021668	multicellular organismal development
**Chd1l **	chromodomain helicase DNA binding protein 1-like	NM_001107704	chromatin remodeling/response to DNA damage stimulus
**RGD1565493 **	similar to DKFZP434I092 protein	XR_007761	Unknown
**Alox15 **	arachidonate 15-lipoxygenase	NM_031010	anti-apoptosis/arachidonic acid metabolic process

**Table 6:** Genes that were Inhibited in the NAcc of LEW rats as Compared with F344 Rats

Gene Symbol	Gene Description	mRNA Accession No.	Biological Process
**Ifi27l **	interferon, alpha-inducible protein 27-like	NM_203410	implantation
**ATP8 **	ATP synthase F0 subunit 8	ENSRNOT00000046201	ion transport (proton transport)
**Igfbp6 **	insulin-like growth factor binding protein 6	NM_013104	regulation of cell growth
**Rad1 **	RAD1 homolog (S. pombe)	NM_001106419	DNA replication
**Dkk3 **	dickkopf homolog 3 (Xenopus laevis)	NM_138519	multicellular organismal development/negative regulator of Wnt signaling pathway
**Igfbp2 **	insulin-like growth factor binding protein 2	NM_013122	regulation of cell growth
**Thrsp **	thyroid hormone responsive	NM_012703	Protein binding (thyroid hormone-induced neuronal cell death)
**LOC684785 **	similar to pleckstrin homology domain-containing, family A (phosphoinositide binding specific) member 2	ENSRNOT00000022097	Unknown
**Slc6a20 **	solute carrier family 6 (neurotransmitter transporter), member 20	NM_133296	neurotransmitter transport
**Ogn **	osteoglycin	NM_001106103	protein binding
